# Beating Standard Quantum Limit with Weak Measurement

**DOI:** 10.3390/e23030354

**Published:** 2021-03-16

**Authors:** Geng Chen, Peng Yin, Wen-Hao Zhang, Gong-Chu Li, Chuan-Feng Li, Guang-Can Guo

**Affiliations:** 1 CAS Key Laboratory of Quantum Information, University of Science and Technology of China, Hefei 230026, China; chengeng@ustc.edu.cn (G.C.); yp123@mail.ustc.edu.cn (P.Y.); zwh5618@ustc.edu.cn (W.-H.Z.); lgc1997@mail.ustc.edu.cn (G.-C.L.); gcguo@ustc.edu.cn (G.-C.G.); 2 CAS Center For Excellence in Quantum Information and Quantum Physics, University of Science and Technology of China, Hefei 230026, China

**Keywords:** weak measurement, Heisenberg scaling, quantum enhanced precision

## Abstract

Weak measurements have been under intensive investigation in both experiment and theory. Numerous experiments have indicated that the amplified meter shift is produced by the post-selection, yielding an improved precision compared to conventional methods. However, this amplification effect comes at the cost of a reduced rate of acquiring data, which leads to an increasing uncertainty to determine the level of meter shift. From this point of view, a number of theoretical works have suggested that weak measurements cannot improve the precision, or even damage the metrology information due to the post-selection. In this review, we give a comprehensive analysis of the weak measurements to justify their positive effect on prompting measurement precision. As a further step, we introduce two modified weak measurement protocols to boost the precision beyond the standard quantum limit. Compared to previous works beating the standard quantum limit, these protocols are free of using entangled or squeezed states. The achieved precision outperforms that of the conventional method by two orders of magnitude and attains a practical Heisenberg scaling up to $n=10^{6}$ photons.

## 1. Introduction

A measurement is normally implemented by coupling a measurement apparatus (MA) to a test quantum system (QS) and detecting the level of meter shift, which is decided by the coupling strength. Thus, any parameter related to the coupling strength can be estimated from the meter shift of the MA. In conventional measurement (CM), the meter shift level is within the range of the maximal eigenvalue of the QS operator, which is (in appropriate units) 1 for spin-1/2 particles. When the coupling is weak, the meter shift is much smaller than the uncertainty of the MA’s observables and is hard to decipher. In this case, one cannot make a precise estimation of the parameter of interest.

In contrast, weak value amplification (WVA) can result in a meter shift beyond the eigenvalue when an adequate post-selection is made on the QS operator $\hat{A}$ after the weak coupling, e.g., the post-selection amplifies the outcome of a measurement on a spin-1/2 particle from 1 to 100 [1]. The degree of this amplification can be quantified by the so-called weak value defined as $A_{w}=\frac{\langle \psi_{f}|\hat{A}|\psi_{i}\rangle }{\langle \psi_{f}|\psi_{i}\rangle }$, where $|\psi_{i}\rangle$ and $|\psi_{f}\rangle$ are the pre-and post-selection states of the QS. When these two state vectors are (nearly) orthogonal, and their overlapping $\langle \varphi |\psi \rangle$ is small, the weak value can significantly exceed the normal eigenvalue and lead to an amplified meter shift to overcome certain environmental disturbances [2,3,4,5,6,7,8,9]. Specifically, the ultra-small transversal shift of a beam can be observed when WVA is introduced [10,11], and the longitudinal optical phase can be precisely measured when an imaginary weak value is explored [12,13]; what is more, the optical Kerr effect in a single-photon level can also be amplified through WVA [14].

Despite this progress in experimentation, a few theoretical works raise serious questions about the overall precision obtained from the post-selected probes [15,16,17]. In general, these theoretical works have appealed to the quantum Fisher information (QFI) in the post-selected probes and conclude that the post-selection can only damage the overall precision compared to the CM [18,19,20]. These debates put WVA in a dilemma: the observed improvement in experiments is undoubtedly true, while the theoretical analysis also seems reasonable, so which one is the true judgment?

Therefore, it is an interesting question to explore the unambiguous advantage of WVA to endow us with unprecedented ability in various measurement tasks. In this review, we will first make an in-depth analysis of the debates between experimentalists and theorists, and then we introduce two advanced weak measurement (WM) protocols [21,22], which beat the standard quantum limit or even approach the Heisenberg limit.

## 2. Standard-Quantum-Limited WM Protocols

In the theoretical analysis of WVA, the achievable precision is normally rigorously cast in terms of Fisher information (FI), which represents the most extractable metrology information, or the lower bound of uncertainty in estimating a parameter with an experimental design [23]. In this sense, the FI quantifies the quality of any particular experimental design. A poorly designed experiment necessarily elicits less FI about the estimated parameter than will a well-designed experiment. Maximizing the FI over all the possible experimental schemes, one can define the so-called QFI as the upper bound of the standard FI. In other words, the full QFI is solely decided by the form of the coupling between QS and MA, and can only be attained with an optimal measurement. We give a brief FI analysis for standard WM and show that WVA fails to attain the full QFI theoretically available.

**Figure 1:**
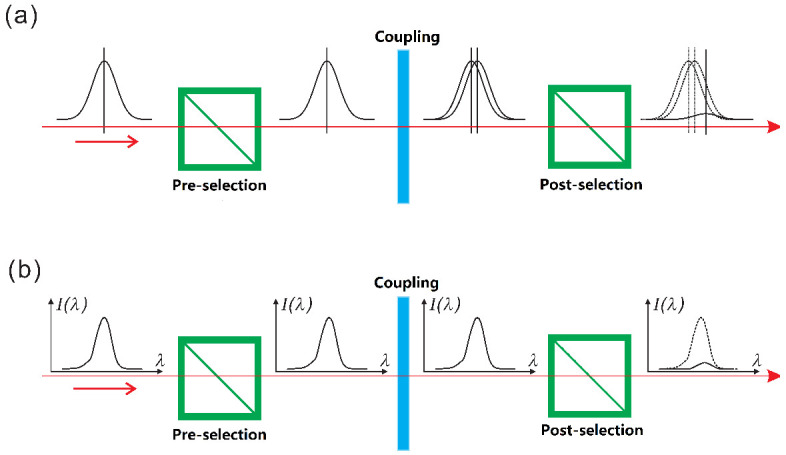
Scheme for a standard weak measurement (WM) protocol. The quantum system (QS) is pre-selected into a superposition of |+1〉 and |−1〉, between which an extra phase is introduced after the coupling with the measurement apparatus (MA). After the post-selection, the two observables of the MA, namely *p* and *q*, are shifted in proportion to the real and imaginary parts of the weak value, as shown in (**a**,**b**), respectively. (Adapted from [12]).

In order to estimate a parameter associated with the coupling between the QS and MA, the QS operator $\hat{A}$ is coupled to the MA operator $\hat{P}$ through the coupling Hamiltonian $H=f\left(t\right)\hat{P}\hat{A}$, where f(t) is a coupling function with a finite support that satisfies ∫f(t)dt=g≪1. Normally, the QS is a two-level system with eigenstates |+1〉 and |−1〉 and the corresponding eigenvalues are +1 and −1. A standard WM protocol can be performed through three procedures as shown in Figure 1, namely, pre- and post-selection of the QS, and a coupling in between. The initial states of the QS and MA are, respectively, $|\psi_{i}\rangle =\cos\left(\theta_{i}/2\right)|-1\rangle +\sin\left(\theta_{i}/2\right)e^{i\phi_{i}}|+1\rangle$ and $|\varphi_{i}\rangle$. After the coupling, The QS and MA evolve into a joint state:

(1) $$|\Psi_{i}\rangle =\cos\left(\theta_{i}/2\right)\left|-1\rangle \right|\varphi_{-}\rangle +\sin\left(\theta_{i}/2\right)e^{i\phi_{i}}\left|+1\rangle \right|\varphi_{+}\rangle ,$$

where $|\varphi_{\pm }\rangle =e^{\mp ig\hat{P}}|\varphi_{i}\rangle$. Without the loss of generality, the MA is normally prepared in a Gaussian superposition state for both of the conjugate variables *p* and *q*, and the corresponding states can be written as

(2) $$|\varphi_{i}\rangle =\int dp{\left(2\pi^{-1}\delta^{2}\right)}^{1/4}\exp\left[-\delta^{2}p^{2}\right]|p\rangle =\int dq{\left(2\pi \delta^{2}\right)}^{-1/4}\exp\left[-\frac{q^{2}}{4\delta^{2}}\right]|q\rangle .$$

The QFI in the joint state described by Equation (1) is calculated as $4\delta^{2}$, which is the upper bound of the elicited FI by any following protocol, including both CM and WM protocols. When a post-selection into $|\psi_{f}\rangle =\cos\left(\theta_{f}/2\right)|-1\rangle +\sin\left(\theta_{f}/2\right)e^{i\phi_{f}}|+1\rangle$ is made on the QS, the MA state is collapsed into $|\varphi_{\surd }\rangle =\cos\left(\theta_{i}/2\right)\cos\left(\theta_{f}/2\right)|\varphi_{-}\rangle +\sin\left(\theta_{i}/2\right)\sin\left(\theta_{f}/2\right)e^{i(\phi_{i}-\phi_{f})}|\varphi_{+}\rangle$ with a probability of $p_{\surd }$, which can be calculated as

(3) $$p_{\surd }=\frac{1+\cos\left(\theta_{i}\right)\cos\left(\theta_{f}\right)+\sin\left(\theta_{i}\right)\sin\left(\theta_{f}\right)\cos\left(\phi_{i}-\phi_{f}\right)e^{-2{\left(g\delta \right)}^{2}}}{2}.$$

When the post-selection fails, the QS is projected into the orthogonal state of $|\psi_{f}\rangle$, and the MA state is collapsed into $|\varphi_{\times }\rangle =\cos\left(\theta_{i}/2\right)\sin\left(\theta_{f}/2\right)|\varphi_{-}\rangle -\sin\left(\theta_{i}/2\right)\cos\left(\theta_{f}/2\right)e^{i(\phi_{i}-\phi_{f})}|\varphi_{+}\rangle$ with a probability of $p_{\times }$ calculated as $p_{\times }=1-p_{\surd }$.

Obviously, the three constituents of post-selection, i.e., the probability $p_{\surd }$, the successful mode $|\varphi_{\surd }\rangle$ and failed mode $|\varphi_{\times }\rangle$, are all related to the coupling strength *g*. Consequently, one can estimate *g* by exploring these three constituents, each of which contains information about *g*. Denoting the QFI of $|\varphi_{\surd }\rangle$ and $|\varphi_{\times }\rangle$ as $Q_{\surd }$ and $Q_{\times }$, respectively, the sum total of the classical and quantum FI contained at different stages of WM can be calculated as $F_{total}=p_{\surd }Q_{\surd }+p_{\times }Q_{\times }+F_{p}$. Specifically, $p_{\surd }Q_{\surd }$ and $p_{\times }Q_{\times }$ represent the maximum FI that can be elicited in the successful and failed modes, and $F_{p}$ stands for the classical FI in the distribution {$p_{\surd }$, 1 − $p_{\surd }$} of the post-selection process.

With the form of $|\varphi_{\left(\surd ,\times \right)}\rangle$ and $p_{\surd }$, $Q_{\surd }$, $Q_{\times }$ and $F_{p}$ can be calculated as

(4) $$F_{p}=\frac{{\left(2\delta \kappa \lambda \right)}^{2}}{p_{\surd }\left(1-p_{\surd }\right)},$$

(5) $$Q_{\surd }=\frac{4\delta^{2}}{p_{\surd }}\left[p_{\surd }+\lambda \left(2\kappa^{2}-1\right)-\frac{{\left(\lambda \kappa \right)}^{2}}{p_{\surd }}\right],$$

(6) $$Q_{\times }=\frac{4\delta^{2}}{1-p_{\surd }}\left[1-p_{\surd }-\lambda \left(2\kappa^{2}-1\right)-\frac{{\left(\lambda \kappa \right)}^{2}}{1-p_{\surd }}\right],$$

where the measurement strength κ is defined as κ=gδ, and λ is expressed as $\lambda =\sin\theta_{i}\sin\theta_{f}\cos\left(\phi_{i}-\phi_{f}\right)e^{-2\delta^{2}g^{2}}$.

The standard WM protocol utilizes solely the successful mode of post-selection, while the other two constituents are normally discarded in the measurement. As a result, only the metrology information in $|\varphi_{\surd }\rangle$ is explored, and in this case we have $F_{SWM}=p_{\surd }Q_{\surd }$. In the weak coupling limit, i.e., the measurement strength κ→0, the total accessible FI by measuring the successful mode is

(7) $$p_{\surd }Q_{\surd }=2\delta^{2}\left[1+\cos\theta_{i}\cos\theta_{f}-\sin\theta_{i}\sin\theta_{f}\cos\left(\phi_{i}-\phi_{f}\right)\right].$$

It can be seen that when either $\theta_{i}=-\theta_{f}$ and $\phi_{i}-\phi_{f}=0$ or $\theta_{i}=\theta_{f}$ and $\phi_{i}-\phi_{f}=\pi$, $F_{SWM}$ achieves the maximum value of $4\delta^{2}$, which saturates the QFI in the joint system-meter state of Equation (1). For other settings of $\left\{\theta_{i},\phi_{i},\theta_{f},\phi_{f}\right\}$, WM can only elicit an amount of FI lower than $4\delta^{2}$; in other words, standard WM normally loses partial information in the joint state since it solely concerns the successful mode of post-selection.

We can conclude that WM cannot enhance the measurement precision in an ideal case from the above calculations. Actually, we can interpret this question more directly, in terms of the signal-to-noise ratio. The WVA can enhance the meter shift (signal) by a factor $∼1/\sqrt{|\langle \varphi_{\times }|\varphi_{\surd }\rangle {|}^{2}}$. Correspondingly, the number of detection events is reduced by a factor of $∼|\langle \varphi_{\times }|\varphi_{\surd }\rangle {|}^{2}$; and thus, the normalized standard error (noise), which is inversely proportional to the square root of the number of detection events, is also amplified by a factor $∼1/\sqrt{|\langle \varphi_{\times }|\varphi_{\surd }\rangle {|}^{2}}$. Considering all these factors, the signal-to-noise ratio stays constant and is not improved via WM.

In contrast, the existing experiments show that WM indeed leads to improved precision, which disagrees with the above theoretical analysis. The major reason for this contradiction is the different situations considered in theory and experiment. The theoretical analysis usually ignores practical constraints, which can be overcome by standard WM to attain better precision in experiment [9,16]. Similar to the signal-to-noise analysis above, we consider a situation where a certain technique’s noise dominates various imperfections in an experiment and generates an error Δ in the meter shift. This technique noise, e.g., the dark current of the detector, or the resolution of a spectrometer, does not change with the number of detection events. Compared to the analysis above, the signal can be amplified due to WVA, while the error remains unchanged before and after the post-selection. Consequently, the signal-to-noise ratio is raised by a factor decided by the weak value. Other practical factors may also confer WM the ability to outperform CM, e.g., when detector saturation occurs, WM maintains a considerable amount of metrology information while detecting an ultra-small fraction of probes. Vaidman proposed the conjecture about this advantage [24], which was then rigorously verified in theory [25] and demonstrated in experiment [26].

Let us return to FI analysis for standard WM. For some special settings of $\left\{\theta_{i},\phi_{i},\theta_{f},\phi_{f}\right\}$, the QFI in the successful mode saturates the QFI in the system–meter joint state, which is calculated as $F_{total}=4\delta^{2}$. The best achievable precision of estimating *g* is given by the Cramér–Rao bound $\Delta g\geq \frac{1}{2\sqrt{N}\delta }$, when the MA consists of *N* non-interacting subsystems. Any additional technique noise inevitably damages the QFI; therefore, the resulted precision is even worse than $\frac{1}{2\sqrt{N}\delta }$ and is undoubtedly subjected to the standard quantum limit. It can be seen that the scaling of the precision is bounded by the QFI in the system–meter state. Although standard WM can saturate this QFI in ideal cases, perhaps outperforming CM in some practical situations, it cannot prompt scaling better than $o(1/\sqrt{N})$.

## 3. Heisenberg Scaling WM Protocols

Scaling is the main issue within quantum metrology, where the goal is to achieve a precision that is inversely proportional to the number of resources (the so-called Heisenberg limit) rather than to the square root of this quantity (which is the standard quantum limit (SQL)). There have been ongoing and significant efforts over the last couple of decades.

The Heisenberg limit is defined as the limit attainable by using all possible quantum resources, and it is normally difficult to saturate in practical measurement tasks. Alternatively, quantum metrology pursues the precision differing from the Heisenberg limit with a small constant-factor before, namely, the Heisenberg scaling. When the constant-factor is small, the Heisenberg scaling beats the SQL with a small number of resources. The relative works attaining Heisenberg scaling generally exploit quantum effects, mainly entanglement [27,28,29,30,31]. A technical issue preventing the application of entanglement to practical measurement is that it is difficult to produce large-size entangled states; therefore, the short-living Heisenberg scaling fails to outperform the conventional method. Besides, the squeezed state also renders precision surpassing the SQL [32,33,34,35], while the limited squeezing ratio prevents it from attaining Heisenberg scaling. Adaptive strategies have been proposed to achieve a standard deviation scaling at the Heisenberg limit, which has been implemented with photons [36], the NV center in diamond [37] and superconducting transmon circuits [38]. Regarding the nonlinear metrology, super-Heisenberg scaling arises from the high-order nonlinear effect itself [39], rather than a technical innovation.

The QFI arising from the coupling determines the scaling of the precision to estimate the coupling strength; therefore, two conditions promise the achievement of Heisenberg scaling, i.e., the QFI is proportional to the square of *N*, and a specific technique to elicit a considerable proportion of this QFI.

We consider a situation in which the overall interaction is related to the number of particles involved in the interaction. The Hamiltonian is expressed as $H=g\hat{A}\hat{n}$, where $\hat{A}$ and $\hat{n}$ represent the number of two-group particles participating in the interaction. A practical example described by this Hamiltonian is the cross-phase modulation between photons, i.e., the Kerr effect between photons. When the task is assigned as measuring the Kerr effect generated by single photons, the QS and MA are pulses containing 1 and *n* photons, respectively. In this measurement, single photons are prepared into a superposition of the eigenstates of $\hat{A}$, i.e., $|\psi_{i}\rangle =|0\rangle +|1\rangle$, where 0, 1 represents the particle number participating in the interaction. Normally this can be achieved by entangling the photon number with another degree of freedom, e.g., preparing the single photons into $|\psi_{i}\rangle =|0\rangle |H\rangle +|1\rangle |V\rangle$ (*V* and *H* represent the vertical and horizontal polarization, respectively). With the polarization as a control qubit, only the *H* polarized component interacts with the strong pulse containing *n* photons, as has been done in [40]. In this experiment, Matsuda et al. observed the Kerr effect in single photon level as low as $10^{-8}$ rad in a photonic crystal fiber. The major factor limiting the precision is the self-phase modulation, since this unexpected effect adds to inherent fluctuation of the strong pulse, which is eventually measured as a probe in this experiment. Taking this self-phase modulation into account, the Hamiltonian is written as $H=g\hat{A}\hat{n}+g_{s}{\hat{n}}^{2}$, where $g_{s}$ is the coupling strength of self-phase modulation.

**Figure 2:**
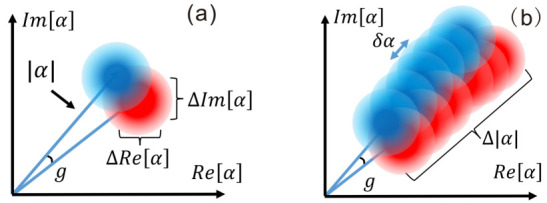
The precision to estimate coupling strength *g* with different probing states. (**a**) Probing with coherent state $|\alpha \rangle$: the precision of measuring a phase Arg(α) is limited by the inherent uncertainty ΔRe[α]=ΔIm[α]=1/2, hence it is standard-quantum-limited. (**b**) Probing with mixed state: the initial state is a statistical ensemble of $|\alpha \rangle$, each of which acquires a phase decided by |α|, and interferes (with itself) with varying probability due to the post-selection. A shift in the radical direction is generated and its level is proportional to the length squared. For a mixed state, the length can be increased to ∼|α|, which leads to Heisenberg scaling precision. (Adapted from [21]).

The interaction strength *g* can be probed with strong pulses in either pure or mixed states, as shown in Figure 2. To determine the best attainable precision in the estimation of *g*, firstly we calculate the QFI in the system–meter state for both pure and mixed probes. Consider some state, pure at this stage, $|\psi \rangle |\varphi \rangle$, where $|\psi \rangle$ is the state of the system, related to $\hat{A}$, and $|\varphi \rangle$ is the state of the meter, related to $\hat{n}$. The QFI is given by

(8) $$\begin{array}{c} I_{q}=4\Delta H^{2}=4\langle \psi |{\hat{A}}^{2}|\psi \rangle \langle \varphi |{\hat{n}}^{2}|\varphi \rangle -4\langle \psi |\hat{A}{|\psi \rangle }^{2}\langle \varphi |\hat{n}{|\varphi \rangle }^{2}=4\langle \psi |{\hat{A}}^{2}|\psi \rangle \Delta n^{2}+4{\Delta A}^{2}\langle \varphi |\hat{n}{|\varphi \rangle }^{2}, \end{array}$$

where $\Delta O^{2}=\langle {\hat{O}}^{2}\rangle -{\langle \hat{O}\rangle }^{2}$ is the variance of an operator with respect to the initial state. Setting $|\psi \rangle$ to an eigenstate of $\hat{A}$ with eigenvalue ξ would yield $I_{q}=4\xi^{2}\Delta n^{2}$, which for coherent states amounts to $I_{q}=4\xi^{2}n$, with $n={\left|\alpha \right|}^{2}$ being the average photon number. For $n^{2}\gg 1$ and ΔA≠0, the other term in $I_{q}$ dominates and we have $I_{q}\approx 4\Delta A^{2}n^{2}$. Note that this does not depend on the particular state $|\varphi \rangle$, i.e., it is valid for coherent states just as much as it is for Fock states. The QFI is bounded by the a limit scaling with $n^{2}$, which allows the best precision beating SQL and attaining Heisenberg scaling.

However, choosing the initial state and showing that it contains sufficient information after the interaction does not ensure that we can extract it efficiently. One should specify how to perform the final measurement and show that indeed the required amount of information can be obtained. To this end, we will show that the standard WM protocol cannot elicit the FI scaling with $n^{2}$ even though it is achievable by the QFI bound. Compared to the method in [40], WM applies a post-selection by projecting the single photons into $|\psi_{f}\rangle =|0\rangle -e^{-i\varepsilon }|1\rangle$. In this case, we obtain a purely imaginary weak value $A_{w}=\frac{\langle \psi_{f}|\hat{A}|\psi_{i}\rangle }{\langle \psi_{f}|\psi_{i}\rangle }=i/\varepsilon$, which means we can estimate *g* by observing the photon number change after the post-selection. For a single probe pulse, we assume the initial state to be a coherent state written as

(9) $$|\alpha \rangle =e^{-{\left|\alpha \right|}^{2}/2}\Sigma_{k}\frac{\alpha^{k}}{\sqrt{k!}}|k\rangle ,$$

where |k〉 denotes the Fock state with photon number as *k*, and the mean photon number of this coherent state is $n={\left|\alpha \right|}^{2}$. The evolution operator is then $U=e^{-iH}=e^{-i(g\hat{A}\hat{n}+g_{s}{\hat{n}}^{2})}$. By replacing $\hat{A}$ with ${\hat{A}}_{w}$, the final state of the probe (before normalization) is

(10) $$|\varphi_{\surd }\rangle \;=e^{\frac{g}{\varepsilon }\hat{n}-ig_{s}{\hat{n}}^{2}}|\alpha \rangle =e^{-\frac{{\left|\alpha \right|}^{2}}{2}+\frac{|\alpha e^{\frac{g}{\varepsilon }}{|}^{2}}{2}-i\frac{g_{s}{\bar{n}}^{2}}{e^{\frac{g}{\varepsilon }}}}|\alpha e^{\frac{g}{\varepsilon }}\rangle .$$

Using $e^{\frac{g}{\varepsilon }}≃1+\frac{g}{\varepsilon }$, we can see that the normalization is given by $e^{{\left|\alpha \right|}^{2}\frac{g}{\varepsilon }}$, which also means that the dependency of the probability for post-selection, on the meter state, is given by

(11) $$p_{\surd }∼e^{{2|\alpha |}^{2}\frac{g}{\varepsilon }}≃1+2{\left|\alpha \right|}^{2}\frac{g}{\varepsilon }.$$

The average photon number for the final state is

(12) $$\langle \hat{n}\rangle ={\left|\alpha \right|}^{2}e^{2\frac{g}{\varepsilon }}≃{\left|\alpha \right|}^{2}\left(1+2\frac{g}{\varepsilon }\right),$$

with the mean photon number change as

(13) $$\Delta n≃{\left|\alpha \right|}^{2}\frac{2g}{\varepsilon }.$$

It can be seen that this mean photon number change acquires an amplification of $\frac{1}{\varepsilon }$ due to the WVA; what is more, the self-modulation item in the Hamiltonian does not contribute to the photon number change. In this case, the self-modulation effect, which harms the measurement precision in [40], can be circumvented here by measuring the photon number change instead of the phase. However, the normalized change $\frac{\Delta n}{{\left|\alpha \right|}^{2}}=\frac{2g}{\varepsilon }$ is approximately in the order of $10^{-6}$, which cannot be detected with currently available techniques since the amplitude resolution of an electrical signal is inversely proportional to the response bandwith. Regarding the scaling of the precision probing with a pure coherent state, of which the quantum fluctuation is ∼|α|, the precision is calculated as

(14) $$\Delta g=\frac{\left|\alpha \right|}{\frac{\partial \Delta n}{\partial g}}=\frac{\varepsilon }{2\sqrt{n}}.$$

Not unexpectedly, the scaling is still standard-quantum-limited with the probe of a coherent state.

In the following section, we show that a phase-interacting scenario with the coupling proportional to the number of interacting particles can provide the QFI, and two modified WM protocols can elicit this QFI to achieve practical Heisenberg scaling precision.

### 3.1. Heisenberg Scaling WM Protocol Probing with Mixed States

In this section we review a modified WM technique to measure the interacting strength of two groups of particles. We will show that by utilizing a mixed probe of various coherent states with significantly different α, the QFI scales with the square of the mean photon number such as that for the pure coherent state; what is more, by observing the mean photon number change in the post-selected pulses, this QFI can be elicited to render an end precision attaining Heisenberg scaling. To show how the mixed probe state works, as usual, we calculate the QFI bound for it. We can extend the QFI calculation for the pure state to mixed states by taking a weighted average of the $I_{q}$. For a statistical mixture of states $|\varphi^{a}\rangle$ with probability $p_{a}$, the convexity of the QFI implies that the QFI of the mixed state, $I_{q}^{m}$, is bounded by $I_{q}^{m}\leq \sum_{a}p_{a}I_{q}^{a}$, where $I_{q}^{a}$ is the QFI in Equation (8) for a state $|\varphi^{a}\rangle$. That is

(15) $$\begin{array}{c} I_{q}^{m}\leq 4\sum_{a}p_{a}\left(\langle \psi |{\hat{A}}^{2}|\psi \rangle \Delta_{a}n^{2}+\Delta A^{2}n_{a}^{2}\right), \end{array}$$

where $n_{a}=\langle \varphi^{a}|\hat{n}|\varphi^{a}\rangle$ is the average photon number of state $|\varphi^{a}\rangle$, $\Delta_{a}n^{2}=\langle {\hat{n}}^{2}\rangle -{\langle \hat{n}\rangle }^{2}$ is the variance for that state. In the case of coherent states, $\frac{n}{\Delta n}=\sqrt{n}$ so when n≫1, the term containing $n_{a}^{2}$ in the QFI dominates and the bound can be approximated by:

(16) $$\begin{array}{c} 4\sum_{a}p_{a}\left(\langle \psi |{\hat{A}}^{2}|\psi \rangle \Delta_{a}n^{2}+\Delta A^{2}{\left(n_{a}\right)}^{2}\right)≃4\sum_{a}p_{a}\Delta A^{2}n_{a}^{2}=4\Delta A^{2}\left(\mathrm{var}\left(n\right)+N^{2}\right), \end{array}$$

where var(n) and *N* are, respectively, the variance and average for the distribution of the photon number of the mixed state. We can see that by mixing coherent states one can increase the variance so that $\mathrm{var}\left(n\right)\propto N^{2}$, giving a bound that is $\propto 2N^{2}$, which improves the upper bound to the QFI.

To show that this mixed probe not only gives rise to QFI of $\propto N^{2}$, we assume $N=={\int d|\alpha |}^{2}P({\left|\alpha \right|}^{2}){\left|\alpha \right|}^{2}$ and its variance ${\int d|\alpha |}^{2}P({\left|\alpha \right|}^{2}){\left({\left|\alpha \right|}^{2}-N^{2}\right)}^{2}=\sigma^{2}$, with $P({\left|\alpha \right|}^{2})$ denoting the weight for each continuous component state $|\alpha \rangle$. Since the probability of post selection depends on the initial state of the meter, the distribution above will change according to $P_{f}∼P({\left|\alpha \right|}^{2})p_{\surd }$. To calculate the average photon number after post-selection we have

(17) $$\begin{array}{cc} \; & N^{\prime }=\frac{{\int d|\alpha |}^{2}P({\left|\alpha \right|}^{2})P_{\surd }{\langle n\rangle }_{\alpha }}{{\int d|\alpha |}^{2}P({\left|\alpha \right|}^{2})P_{\surd }}\\ \; & =\frac{{\int d|\alpha |}^{2}P({\left|\alpha \right|}^{2})(1+{2|\alpha |}^{2}\frac{g}{\epsilon }){\left|\alpha \right|}^{2}(1+\frac{2g}{\epsilon })}{{\int d|\alpha |}^{2}P({\left|\alpha \right|}^{2})(1+{2|\alpha |}^{2}\frac{g}{\epsilon })}\\ \; & =\frac{\left(N+2\frac{g}{\epsilon }(\sigma^{2}+N^{2}))(1+\frac{2g}{\epsilon }\right)}{1+2N\frac{g}{\epsilon }}\\ & ≃N+2\frac{g}{\epsilon }(\sigma^{2}+N), \end{array}$$

where in the last step we use $\frac{1}{1+x}≃1-x$ and drop any orders higher than 1 in g/ε.

From the above equation, the mean photon number change is

(18) $$\delta N_{mixed}≃2\frac{g}{\epsilon }(\sigma^{2}+N).$$

Compared to the mean photon number change when probing with a coherent state, an extra item $\frac{2g}{\varepsilon }\sigma^{2}$ appears here that greatly boosts the change of mean photon number. Since the mixed state can be generated through the amplitude-modulation of a certain coherent state $|\alpha_{0}\rangle$, the standard deviation σ exceeds quantum fluctuation $\left|\alpha_{0}\right|$ and reaches a level of σ∝N. Correspondingly, the estimation precision of *g* with ν probe pulses is calculated as

(19) $$\Delta g≃\frac{\sigma }{\sqrt{\nu }\frac{\partial \frac{2g\sigma^{2}}{\varepsilon }}{\partial g}}\propto \frac{\varepsilon }{2N\sqrt{\nu }},$$

Considering $\nu ∼\tilde{\nu }{\left|\langle \psi_{f}|\psi_{i}\rangle \right|}^{2}∼\frac{\tilde{\nu }\varepsilon^{2}}{4}$ with $\tilde{\nu }$ denoting the number of incident pulses, the scaling of the precision is

(20) $$\Delta g\propto \frac{1}{\sqrt{\tilde{\nu }}N},$$

which is independent of the post-selection probability. By defining *N* as the number of used resources, it can be seen that when the probe is a mixture of various coherent states, the precision in estimating *g* can beat the SQL and attain the Heisenberg scaling.

To show the achieved scaling with *N* by observing the photon number change in the post-selected pulses, we can also calculate the classical Fisher information as

(21) $$\begin{array}{c} I_{c}=\nu \langle {\left(\frac{\partial \ln P_{f}}{\partial g}\right)}^{2}\rangle =4\nu \frac{\sigma^{2}}{\varepsilon^{2}}\propto \tilde{\nu }N^{2}, \end{array}$$

which indicates a practical Heisenberg scaling precision.

**Figure 3:**
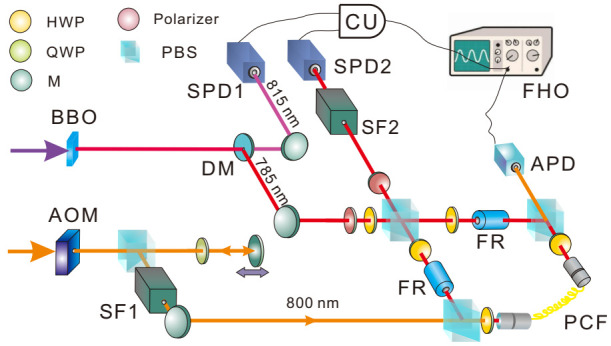
Setup of estimating single photon Kerr effect with mixed probe state. Single photons of 785 nm interact with strong pulses of 800 nm in the photonic crystal fiber (PCF). The amplitude of the strong pulses is modulated to generate a mixture with different coherent states. Post-selecting the single photons and detecting the intensity of corresponding strong pulses with a full HD oscilloscope, the coupling strength *g* can be estimated from the mean photon number shift of the strong pulses. The achieved precision Δg is inversely proportional to the mean photon number *N*, which is the so-called Heisenberg scaling. (Adapted from [21]).

In the calculation of classical Fisher information, we merely focus on the post-selected pulses, and discard others failing the post-selection. In general, the post-selection is rare. This can diminish the precision due to a decrease in the number of successful post-selecting events $\nu ∼\tilde{\nu }{\left|\langle \psi_{f}|\psi_{i}\rangle \right|}^{2}\approx \frac{\tilde{\nu }\varepsilon^{2}}{4}$. However, due to the weak value amplification, one obtains another amplification factor of $\epsilon^{-2}$ on the meter shift. Consequently, by calculating the Fisher information, the dependence on the post-selection probability cancels. It is somewhat surprising that although both pure and mixed probes generate QFI scaling with $N^{2}$, by measuring the meter shift of the successful mode of post-selection, only a mixed probe renders Heisenberg scaling in the estimation of *g*. An experimental work applies this method to measure the optical Kerr effect in a single photon level [21], with the setup shown in Figure 3.

The two interacting parties are heralded single photons from a spontaneous parametric down-conversion process, and classical strong pulses from a femtosecond laser. The mixed coherent states are generated after modulating strong pulse amplitude by an acoustic optical modulator (AOM), and the pulse intensity varies with time to form a mixed state for which the fluctuation amplitude (standard deviation) is ∝N. The single photon state is set as $(|V\rangle +\left|H\rangle \right)/\sqrt{2}$ (*V* and *H* represent the vertical and horizontal polarization, respectively). After entering the polarization Sagnac interferometer, only the *V* component is synchronized with the strong pulses in the photonic crystal fiber to generate a single photon Kerr effect; therefore, the system becomes $|\psi_{i}\rangle =\left({|1\rangle }_{V}+{|0\rangle }_{H}\right)/\sqrt{2}$, where {0,1} represents the interacting photon number. The interacting Hamiltonian is expressed as $H=g\hat{A}\hat{n}$, in which $\hat{A}=\left|H\rangle \langle H\right|+\left|V\rangle \langle V\right|$.

**Figure 4:**
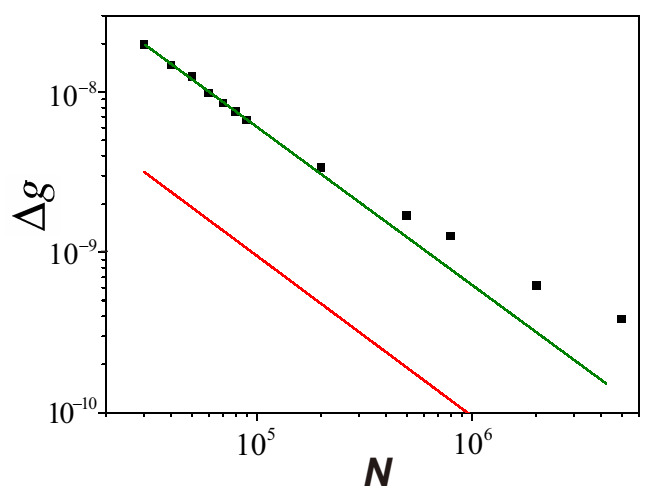
A practical Heisenberg scaling in experiment.The estimation precision of *g* against the mean photon number *N* of the mixed state is plotted. For $N<10^{5}$, the precision follows a Heisenberg scaling of $\Delta g≃6.3\times 10^{-4}N^{-1}$ rad, shown as a green line, obtained by fitting these points. The red line is a bound on the precision for mixed states, taking account of the QFI for each member in the ensemble, given by $\Delta g_{\min}≃0.95\times 10^{-4}N^{-1}$ rad. (Adapted from [21]).

The measurement is performed by post-selecting the single photons in $|\psi_{f}\rangle =\left(|V\rangle -e^{-i\epsilon }|H\rangle \right)/\sqrt{2}$, and using the successful events to trigger a full HD oscilloscope that records the intensity of corresponding strong pulses. This full HD oscilloscope provides a resolution of 1/4096 of the amplitude, and the mean photon number change can be exactly detected. Through analyzing the mean intensity of these post-selected strong pulses, we can estimate *g* through the equation $\delta N_{mixed}≃\frac{2g\sigma^{2}}{\epsilon }$. The measurement precision is estimated from Equation (19), and by plotting the precision against *N*, an evident Heisenberg scaling is observed as shown in Figure 4. The attained Heisenberg scaling maintains up to $N∼10^{5}$ photons and the ultimate precision outperforms that in [40] by one order of magnitude.

To summarize, by mixing a series of pure states, the uncertainty of the probe state is increased, whereas the end uncertainty in the estimation of *g* is minimized. What is more, this estimation uncertainty vanishes linearly with the mean photon number *N* in the probe; and thus, a practical Heisenberg scaling precision is attained. This method is either entanglement or squeezing free, and the relying quantum resource is the single photon superposition, which is easy to generate and operate. In this scheme, the imaginary weak value is explored, and hence the photon number change is measured instead of measuring the phase (real weak value). On one hand, this is a technique issue since it is easier to implement photon counting rather than measuring an ultra-small phase shift directly. On the other hand, in order to achieve the Heisenberg scaling, a mixed probe state is required. A mixed state in a photon number is easy to produce by modulating the intensity of laser pulses with an acoustic–optical modulator. Furthermore, the self-modulation effect, which harms the measurement precision of the phase, can be circumvented here by measuring the photon number.

It is worth noting that both the theoretical calculations and the experimental results are based on the approximation expanding exp(g/ε) to the first order in *g*. In experiment, the smallest ε is decided by the extinction ratio of the polarizer used for post-selection, which is normally in the order of $10^{-2}$, while *g* is in the order of $10^{-8}$. As a result, we always have g/ε≪1 with the current experimental setup. In this sense, the ability to further reduce ε necessarily implies the possibility to measure smaller *g*, without diminishing the end precision.

### 3.2. Heisenberg Scaling WM Protocol by Measuring the Post-Selection Probability

Regarding the FI calculation for a pure state probe, the QFI is $∼n^{2}$ while it cannot be elicited by a standard WM protocol. A natural question is this: is it possible to make full use of this QFI to attain the Heisenberg scaling with a proper protocol? To answer this question, we return to the FI analysis of the three constituents in a standard WM protocol.

For a WM protocol with the pre- and post-selected states as $|\psi_{i}\rangle =|0\rangle +|1\rangle$ and $|\psi_{f}\rangle =|0\rangle -e^{-i\varepsilon }|1\rangle$, respectively, the interaction strength *g* can be estimated by exploring the three constituents, i.e., the post-selection probability, the successful and failure modes in post-selection. The corresponding FI contained in these three constituents is calculated as

(22) $$\begin{array}{cc} \; & F_{p}=n^{2}, \end{array}$$

(23) $$\begin{array}{cc} \; & P_{d}Q_{d}=\left(1-\varepsilon^{2}/4\right)n, \end{array}$$

(24) $$\begin{array}{cc} \; & P_{r}Q_{r}=\varepsilon^{2}n/4, \end{array}$$

and the successful post-selection probability is

(25) $$P_{\surd }=\frac{1-\cos(gn+\varepsilon )}{2}.$$

**Figure 5:**
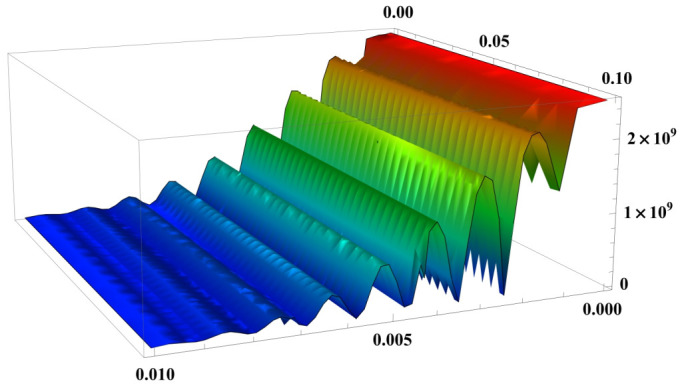
The classical information $F_{p}$ contained in the post-selection process. $F_{p}$ is calculated for varying interaction strength *g* and post-selection parameter ε, when the mean photon number of strong pulses is $n=5\times 10^{4}$. As g→0, $F_{p}$ becomes dominant in $F_{tot}$ and scales with $n^{2}$, which means a practical Heisenberg scaling precision is attainable by measuring the successful post-selection probability. (Adapted from [22]).

Considering the two parties involved in the interaction, i.e., the strong pulses consisting of a substantial amount of photons and the single photon pulses, in principle both of these two parties can be measured to estimate the interaction strength between them. Intuitively, the strong pulses are the primary choice to be measured since more photons usually imply more metrology information. From this point of view, [14,40] measure the phase shift of strong pulses and observe the Kerr effect in a single photon level; however, the precision achieved in these two works is still standard-quantum-limited. Despite containing only a single photon, single photon pulses provide a quantum-enhanced FI of $F_{p}=n^{2}$, which can be elicited by simply measuring the successful post-selection probability of the single photons. Conversely, strong pulses containing *n* photons only provide a negligible amount of FI, hence can be completely discarded after the interaction with the single photons. Figure 5 shows the calculated $F_{p}$ against the post-selection parameter ε and the interaction strength *g*. It can be seen that for an ultra-small *g* and a small value of ε, $F_{p}$ is approximately equal to $n^{2}$, which suggests a Heisenberg scaling precision of estimating *g*.

The achievable precision can also be predicted from the ratio of the uncertainty on the meter and the sensitivity of the meter shift to *g*. In this case, the meter is selected as the successful post-selection probability of single photons. Specifically, the sensitivity is calculated as $s=\frac{\partial p_{\surd }}{\partial g}=n\sin\left(gn+\varepsilon \right)/2≃n\left(gn+\varepsilon \right)/2$ when (gn+ε)≪1, and in this case the uncertainty of the measured $p_{\surd }$ is $\Delta p_{\surd }={\left(p_{\surd }\right)}^{1/2}≃\left(gn+\varepsilon \right)/2$. The precision is calculated as

(26) $$\Delta g≃\frac{\Delta p_{\surd }}{s}=\frac{1}{n}.$$

By defining *n* as the used resources, this expression implies the accessibility of Heisenberg-limited precision with this scheme.

**Figure 6:**
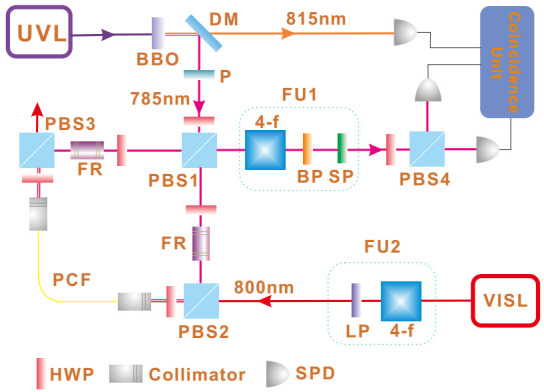
Setup for estimating single photon Kerr effect by measuring the post-selection probability. The 815 nm photons serve as triggers and the heralded 785 nm photons interact with strong pulses (800 nm) in an 8 m long photonic crystal fiber (PCF), centering in a polarization Sagnac interferometer (PSI). The interaction strength *g* is estimated from the distribution of successful and failed post-selection probabilities. (Adapted from [22]).

**Figure 7:**
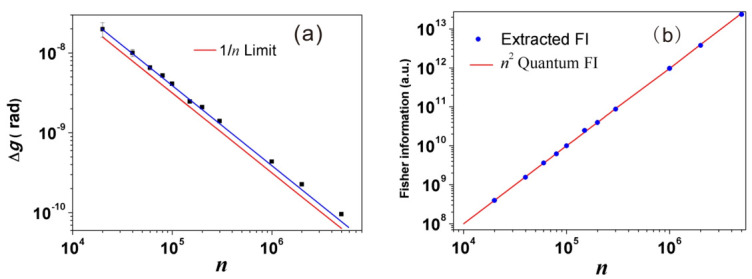
A practical Heisenberg scaling approaching the Heisenberg limit.(**a**) Experimental verification of Heisenberg-limited precision. The achieved precision shows good agreement with the 1/n fitting line up to $n=10^{6}$ photons, and an ultimate precision of $∼10^{-10}$ rad is obtained. (**b**) The amount of extracted Fisher information (FI) for different *n*. The $n^{2}$ scaling also indicates that the Heisenberg limit is approached in this measurement. (Adapted from [22]).

An experimental demonstration of this modified WM protocol is implemented in [22], with the setup shown in Figure 6. The two interacting parties are uniform laser pulses in a coherent state and heralded single photons from a spontaneous parametric down-conversion process. The single photons separate into an equal superposition of clockwise and counter-clockwise propagation at the entrance of the polarization Sagnac interferometer (PSI), and the horizontally polarized strong pulses from an 800 nm visible laser (VISL) are coupled into PSI by PBS2. The strong pulses are synchronized and collinear with 785 nm single photons, then they are coupled into the photonic crystal fiber (PCF). Only the clockwise component of the single photons can interact with the strong pulse; hence, the photon number state becomes $|\psi_{i}\rangle =\left({|1\rangle }_{H}+{|0\rangle }_{V}\right)/\sqrt{2}$, where {0,1} represents the interacting photon number. The photon polarization is maintained after the PCF, and the birefringence is eliminated by two Faraday units, each consisting of a 45${}^{\circ }$ Faraday rotator and an HWP. After exiting the PCF, strong pulses depart the PSI from PBS3 and two counter-propagating components of single photons are finally combined at PBS1. Eventually, single photons are projected into $|\psi_{f}\rangle =|H\rangle +e^{i(\pi -\varepsilon )}|V\rangle$. The rates of accepted and rejected events are simultaneously recorded to calculate the successful post-selection probability $p_{\surd }$. By measuring $p_{\surd }$ for different values of *g*, the sensitivity *s* can be determined as the slope of the fitting line. The uncertainty in the meter is determined as the standard deviation $\sigma_{\nu }$ for ν times measurement of $p_{\surd }$. The achieved precision is defined as the ratio of $\sigma_{\nu }$ and *s*, and the results shown in Figure 7a are consistent with the theoretical prediction in Equation (26).

With the measured $P_{d}$ and *s*, the extracted classical FI can be calculated as [20]

(27) $$F_{p}=\frac{1}{P_{d}}{\left(\frac{\partial P_{d}}{\partial g}\right)}^{2}+\frac{1}{1-P_{d}}{\left(\frac{\partial (1-P_{d})}{\partial g}\right)}^{2},$$

and the results are shown in Figure 7b. The classical FI in the experiment is all located on the line of $n^{2}$, implying the practical precision nearly saturates the QFI in the system–meter joint state.

As the WM protocol utilizes a mixed probe, only single photon superposition is required here to achieve the Heisenberg scaling, without the usage of entanglement or squeezing. Furthermore, the measurement relies on single photon counting, which is easy to implement in experiment. Another major advantage is that the self-phase modulation of the strong pulses is insignificant here, since the strong pulses are discarded and only the single photons are measured finally.

## 4. The Implementation of the Scheme in a Ramsey-Type Model with Qubits

In order to describe our scheme in a more general context, it is meaningful to apply the weak measurement proposals into qubit–qubit system [41]. In this section, we will show these two methods can also be applied to a Ramsey-type model with qubits, such as the interaction between photons and atoms.

When a single spin (system) is coupled to an ensemble of other *N* spins (probes), the interaction Hamiltonian is expressed as $H=g{\hat{\sigma }}_{z}{\hat{S}}_{z}$, where ${\hat{\sigma }}_{z}$ (${\hat{S}}_{z}=\sum_{i=1}^{N}\sigma_{z}^{i}$) is system (probe) operator, respectively. To estimate *g*, normally one measures the phase that the pure probes accumulate due to the interaction; however, this method is limited by the SQL, $\Delta g∼1/\sqrt{N}$. We will show with the two modified WM protocols introduced in this review, that the achieved precision is significantly improved from SQL to Heisenberg scaling. First we consider a single pure probe state $|\varphi^{a}\rangle$ with the mean photon number as *n*, where the single spin is prepared in a state $|\psi_{i}\rangle$ and after the interaction is post-selected to $|\psi_{f}\rangle$.

Assuming that gn≪1, the joint system–meter state after coupling is

(28) $$|\chi \rangle ≃\left(I-ig\sigma_{z}⊗S_{z}\right)|\psi_{i}\rangle |\varphi^{a}\rangle ,$$

The post-selection leads to the final state of the probe as

(29) $$|\varphi_{f}^{a}\rangle ≃\frac{1}{\sqrt{p_{\surd }}}\langle \psi_{f}|\chi \rangle ≃\frac{\langle \psi_{f}|\psi_{i}\rangle }{\sqrt{p_{\surd }}}e^{-ig{\left(\sigma_{z}\right)}_{w}S_{z}}|\varphi^{a}\rangle ,$$

These standard operations in a weak measurement result in a purely imaginary weak value expressed as ${\left(\sigma_{z}\right)}_{w}=\frac{\langle \psi_{f}|\sigma_{z}|\psi_{i}\rangle }{\langle \psi_{f}|\psi_{i}\rangle }=i/\varepsilon$.

The post-selection probability is calculated as

(30) p√=Tr(ψfψf⊗Ipχχ)=|ψf|ψi|24(1+2gIm(σz)wφaSzφa+g2n2ε2),

where $I_{p}$ is the identity matrix operating on the probes’ subspace. In the case that $|\varphi^{a}\rangle$ is a coherent state with mean photon number *n*, the post-selection probability to obtain the post-selected state $|\varphi_{f}^{a}\rangle$ is

(31) $$\begin{array}{ccc} p_{\surd } & = & {\left(\frac{gn+\varepsilon }{2}\right)}^{2}, \end{array}$$

which is exactly consistent with the result in Equation (25). Combining with the analysis in Section 3.2, it is possible to attain the Heisenberg scaling in this experimental scenario by measuring the post-selection probability of the single spin.

Otherwise, one can using a mixed probe state $\rho_{p}=\sum_{a}p_{a}|\varphi^{a}\rangle \langle \varphi^{a}|$, where $p_{a}$ denotes the weight of $|\varphi^{a}\rangle$ in this ensemble of spins and the mean photon number of this mixed state is *N*. In this case, the interaction leads to a system–meter state

(32) $$\rho ≃\left(I-ig\sigma_{z}⊗S_{z}\right)|\psi_{i}\rangle \langle \psi_{i}|⊗\rho_{p}\left(I+ig\sigma_{z}⊗S_{z}\right),$$

and the post-selection results in the final state of the probes

(33) $$\rho_{p}^{f}≃\frac{1}{N_{\rho }}\sum_{a}p_{a}p_{\surd }|\varphi_{f}^{a}\rangle \langle \varphi_{f}^{a}|,$$

The probability of obtaining $|\varphi_{f}^{a}\rangle$ is the product of the weight in the ensemble and the post-selection probability of $|\varphi^{a}\rangle$, expressed as $p_{a}p_{\surd }$. By summing up these probabilities over *a*, the re-normalization $N_{\rho }$ is calculated as $N_{\rho }=\sum_{a}p_{a}p_{\surd }$. Then the observable $S_{z}$ appearing in the Hamiltonian is measured, resulting in

(34) 〈Sz〉=Tr[ρpfSz]≃1NρTr∑apa|ψf|ψi|2(I+gIm(σz)w⊗Sz)φaφa(I+gIm(σz)w⊗Sz)Sz.

Keeping only terms of first order in *g* we have that

(35) 〈Sz〉≃1Nρ∑apa|ψf|ψi|2φaSzφa+2gIm(σz)wφaSz2φa.

Substituting $p_{\varphi |a}$ and $N_{\rho }$ into the above equation, and canceling the items of high order in *g*, we conclude that

(36) 〈Sz〉≃∑apa|ψf|ψi|2φaSzφa+2gIm(σz)wφaSz2φa∑apa|ψf|ψi|21+2gIm(σz)wφaSzφa=〈Sz〉0+2gIm(σz)w〈Sz2〉01+2gImσw〈Sz〉0≃Sz0+2gIm(σz)w〈Sz2〉0−〈Sz〉02,

where ${\langle •\rangle }_{0}$ means a weighted average over *a* with regard to $\rho_{p}$ before the interaction. For the modified state 〈Sz〉≃〈Sz〉0+2gIm(σz)w〈Sz2〉0−〈Sz〉02. This yields an estimation for *g* with a precision of $\Delta g=\frac{\Delta \langle S_{z}\rangle }{|\frac{d\langle S_{z}\rangle }{dg}|}∼\frac{1}{\Delta H}$. Combining with the analysis in Section 3.1, when the probe is in a mixed state with ΔH∼N, i.e., $\rho =\frac{1}{2}\left(\prod_{i=1}^{N}|{↑}_{z}^{i}\rangle \langle {↑}_{z}^{i}|+\prod_{i=1}^{N}|{↓}_{z}^{i}\rangle \langle {↓}_{z}^{i}|\right)$, one can attain the Heisenberg scaling in the estimation of *g*.

## Data Availability

The authors declare that all data supporting the findings of this study are available within the article from the corresponding author upon reasonable request.

## References

[1] Aharonov Y., Albert D.Z., Vaidman L. (1988). How the result of a measurement of a component of the spin of a spin-1/2 particle can turn out to be 100. Phys. Rev. Lett..

[2] Strübi G., Bruder C. (2013). Measuring Ultrasmall Time Delays of Light by Joint Weak Measurements. Phys. Rev. Lett..

[3] Magana-Loaiza O.S., Mirhosseini M., Rodenburg B., Boyd R.W. (2014). Amplification of Angular Rotations Using Weak Measurements. Phys. Rev. Lett..

[4] Pang S., Brun T.A. (2015). Improving the Precision of Weak Measurements by Postselection Measurement. Phys. Rev. Lett..

[5] Pang S., Alonso J.R.G., Brun T.A., Jordan A.N. (2016). Protecting weak measurements against systematic errors. Phys. Rev. A.

[6] Kedem Y. (2012). Using technical noise to increase the signal-to-noise ratio of measurements via imaginary weak values. Phys. Rev. A.

[7] Starling D.J., Dixon P.B., Jordan A.N., Howell J.C. (2009). Optimizing the signal-to-noise ratio of a beam-deflection measurement with interferometric weak values. Phys. Rev. A.

[8] Nishizawa A., Nakamura K., Fujimoto M.-K. (2012). Weak-value amplification in a shot-noise-limited interferometer. Phys. Rev. A.

[9] Jordan A.N., Martinez-Rincon J., Howell J.C. (2014). Technical Advantages for Weak-Value Amplification: When Less Is More. Phys. Rev. X.

[10] Hosten O., Kwiat P. (2008). Observation of the spin Hall effect of light via weak measurements. Science.

[11] Dixon P.B., Starling D.J., Jordan A.N., Howell J.C. (2009). Ultrasensitive beam deflection measurement via interferometric weak value amplification. Phys. Rev. Lett..

[12] Xu X.-Y., Kedem Y., Sun K., Vaidman L., Li C.-F., Guo G.-C. (2013). Phase estimation with weak measurement using a white light source. Phys. Rev. Lett..

[13] Bruuner N., Simon C. (2010). Measuring small longitudinal phase shifts: Weak measurements or standard interferometry?. Phys. Rev. Lett..

[14] Hallaji M., Feizpour A., Dmochowski G., Sinclair J., Steinberg A.M. (2017). Weak-value amplification of the nonlinear effect of a single photon. Nat. Phys..

[15] Ferrie C., Combes J. (2014). Weak Value Amplification is Suboptimal for Estimation and Detection. Phys. Rev. Lett..

[16] Knee G.C., Gauger E.M. (2014). When Amplification with Weak Values Fails to Suppress Technical Noise. Phys. Rev. X.

[17] Dressel J., Malik M., Miatto F.M., Jordan A.N., Boyd R.W. (2014). Colloquium: Understanding quantum weak values: Basics and applications. Rev. Mod. Phys..

[18] Combes J., Ferrie C., Jiang Z., Caves C.M. (2014). Quantum limits on postselected, probabilistic quantum metrology. Phys. Rev. A.

[19] Tanaka S., Yamamoto N. (2013). Information amplification via postselection: A parameter-estimation perspective. Phys. Rev. A.

[20] Zhang L.-J., Datta A., Walmsley I.A. (2015). Precision metrology using weak measurements. Phys. Rev. Lett..

[21] Chen G., Aharon N., Sun Y.-N., Zhang Z.-H., Zhang W.-H., He D.-Y., Tang J.-S., Xu X.-Y., Kedem Y., Li C.-F. (2018). Heisenberg-scaling measurement of the single-photon Kerr non-linearity using mixed states. Nat. Commun..

[22] Chen G., Zhang L., Zhang W.-H., Peng X.-X., Xu L., Liu Z.-D., Xu X.-Y., Tang J.-S., Sun Y.-N., He D.-Y. (2018). Achieving Heisenberg-scaling precision with projective measurement on single photons. Phys. Rev. Lett..

[23] Jaynes E.T., Bretthorst G.L. (2003). Probability Theory: The Logic of Science.

[24] Vaidman L. (2017). Weak value controversy. Philos. Trans. R. Soc. Math. Phys. Eng. Sci..

[25] Harris J., Boyd R.W., Lundeen J.S. (2017). Weak Value Amplification Can Outperform Conventional Measurement in the Presence of Detector Saturation. Phys. Rev. Lett..

[26] Xu L., Liu Z., Datta A., Knee G.C., Lundeen J.S., Lu Y.Q., Zhang L. (2020). Approaching Quantum-Limited Metrology with Imperfect Detectors by Using Weak-Value Amplification. Phys. Rev. Lett..

[27] Bollinger J.J., Itano W.M., Winel D.J., Heinzen D.J. (1996). Optimal frequency measurements with maximally correlated states. Phys. Rev. A.

[28] Walther P., Pan J.-W., Aspelmeyer M., Ursin R., Gasparoni S., Zeilinger A. (2004). De Broglie wavelength of a non-local fourphoton state. Nature.

[29] Afek I., Ambar O., Silberberg Y. (2010). High-NOON states by mixing quantum and classical light. Science.

[30] Giovannetti V., Lloyd S., Maccone L. (2004). Quantum-Enhanced Measurements: Beating the Standard Quantum Limit. Science.

[31] Pezze L., Smerzi A., Oberthaler M.K., Schmied R. (2018). Philipp Treutlein Quantum metrology with nonclassical states of atomic ensembles. Rev. Mod. Phys..

[32] Goda K., Miyakawa O., Mikhailov E.E., Saraf S., Adhikari R., McKenzie K., Ward R., Vass S., Weinstein A.J., Mavalvala N. (2008). A quantum-enhanced prototype gravitationalwave detector. Nat. Phys..

[33] Grangier P., Slusher R., Yurke B., LaPorta A. (1987). Squeezedlight–enhanced polarization interferometer. Phys. Rev. Lett..

[34] Xiao M., Wu L.-A., Kimble H.J. (1987). Precision measurement beyond the shot-noise limit. Phys. Rev. Lett..

[35] Treps N., Grosse N., Bowen W.P., Fabre C., Bachor H.-A., Lam P.K. (2003). A quantum laser pointer. Science.

[36] Higgins B.L., Berry D.W., Bartlett S.D., Wiseman H.M., Pryde G.J. (2007). Entanglement-free Heisenberg-limited phase estimation. Nature.

[37] Bonato C., Blok M.S., Dinani H.T., Berry D.W., Markham M.L., Twitchen D.J., Hanson R. (2016). Optimized quantum sensing with a single electron spin using real-time adaptive measurements. Nat. Nanotechnol..

[38] Danilin S., Lebedev A.V., Vepsalainen A., Lesovik G.B., Blatter G., Paraoanu G.S. (2018). Quantum-enhanced magnetometry by phase estimation algorithms with a single artificial atom. NPJ Quantum Inf..

[39] Napolitano M., Koschorreck M., Dubost B., Behbood1 N., Sewell R.J., Mitchell M.W. (2007). Interaction-based quantum metrology showing scaling beyond the Heisenberg limit. Nature.

[40] Matsuda N., Shimizu R., Mitsumori Y., Kosaka H., Edamatsu K. (2008). Observation of optical-fibre Kerr nonlinearity at the single-photon level. Nat. Photonics.

[41] Paraoanu G.S. (2011). Generalized partial measurements. Euro. Phys. Lett..

